# Anti-cancer agents in Saudi Arabian herbals revealed by automated high-content imaging

**DOI:** 10.1371/journal.pone.0177316

**Published:** 2017-06-13

**Authors:** Dina Hajjar, Stephan Kremb, Salim Sioud, Abdul-Hamid Emwas, Christian R. Voolstra, Timothy Ravasi

**Affiliations:** 1 KAUST Environmental Epigenetics Program, King Abdullah University of Science and Technology (KAUST), Thuwal, Saudi Arabia; 2 Red Sea Research Center, King Abdullah University of Science and Technology (KAUST), Thuwal, Saudi Arabia; 3 Analytical Core Laboratory, King Abdullah University of Science and Technology (KAUST), Thuwal, Saudi Arabia; 4 NMR Core Laboratory, King Abdullah University of Science and Technology (KAUST), Thuwal, Saudi Arabia; Institute of medical research and medicinal plant studies, CAMEROON

## Abstract

Natural products have been used for medical applications since ancient times. Commonly, natural products are structurally complex chemical compounds that efficiently interact with their biological targets, making them useful drug candidates in cancer therapy. Here, we used cell-based phenotypic profiling and image-based high-content screening to study the mode of action and potential cellular targets of plants historically used in Saudi Arabia’s traditional medicine. We compared the cytological profiles of fractions taken from *Juniperus phoenicea* (Arar), *Anastatica hierochuntica* (Kaff Maryam), and *Citrullus colocynthis* (Hanzal) with a set of reference compounds with established modes of action. Cluster analyses of the cytological profiles of the tested compounds suggested that these plants contain possible topoisomerase inhibitors that could be effective in cancer treatment. Using histone H2AX phosphorylation as a marker for DNA damage, we discovered that some of the compounds induced double-strand DNA breaks. Furthermore, chemical analysis of the active fraction isolated from *Juniperus phoenicea* revealed possible anti-cancer compounds. Our results demonstrate the usefulness of cell-based phenotypic screening of natural products to reveal their biological activities.

## Introduction

Cancer is a leading cause of morbidity and mortality worldwide, exceeding the number of cases of illness and death due to HIV/AIDS, malaria, and tuberculosis [1]. In 2012, 8.2 million cancer-related deaths were recorded and approximately 14 million more cases were diagnosed. The incidence of cancer is predicted to increase to as many as 22 million cases per year over the next 20 years [2]. The pathology of cancer derives from cellular hyperproliferation that mediates cell differentiation, apoptosis, cell growth and invasion, and angiogenesis and metastasis [1, 3, 4]. As both the population and incidence of cancer increase, so too will its economic burden on society. Anti-cancer therapeutic compounds derived from natural products could help to alleviate this burden because such products are structurally complex chemical compounds that efficiently interact with their biological targets.

High-content screening (HCS) optimizes the discovery of biologically active small molecules and their subsequent development into therapeutic compounds by the use of automated microscopy in conjunction with image analysis. A high-throughput platform based on image-processing software that reviews images from automated fluorescence microscopy [5–10], HCS enhances phenotypic profiling by characterization of cells imaged by fluorescence cytology and high-throughput analysis of a broad spectrum of biological attributes. Rapidly established as an efficient methodology for compound screening, HCS is a key technology in drug discovery because it prompts the investigation of changes in cell localization, intensity, texture or shape and hence allows the elucidation of discreet and physiologically applicable cellular processes such as cell or protein movement, morphological changes or protein modification [9, 11]. In addition, mass spectrometry (MS) and nuclear magnetic resonance (NMR) spectroscopy are powerful analytical tools commonly used in the study of the chemical compositions of samples [12–15]. These techniques are often coupled to identify and quantify bioactive molecules in natural products with potential medicinal value [16–20]. High-performance liquid chromatography (HPLC) coupled with NMR spectroscopy has been used in a wide range of natural product studies, particularly to identify the chemical compositions of plant extracts [21, 22].

In this study, we used HCS, MS, NMR and HPLC to investigate the bioactivity of fractions obtained from a selection of plants endemic to the Arabian peninsula. We collected three plants, *Anastatica hierochuntica* (Kaff Maryam), *Juniperus phoenicea* (Arar), and *Citrullus colocynthis* (Hanzal), that have been used in traditional medicine in Saudi Arabia. Previous reports reported anti-cancer activity in *Juniperus phoenicea* and *Citrullus colocynthis* [23–25]. Using cytological profiles of small molecules known to have biological effects and clustering them with the plant fractions, we selected fractions that we expected would perturb human cancer cells and fractions that clustered with known drugs with anti-cancer mechanisms. Knowing that Topoisomerase II (topo II) is a key enzyme that modulates the topology of DNA by transferring an intact DNA duplex through a DNA helix that has been cut during ATP hydrolysis and that topo II is an essential element of the chromosomal scaffold, we then tested the fractions to determine if they inhibited topoisomerase. Furthermore, we analyzed the active fractions of *J*. *phoenicea* using liquid chromatography-MS (LC-MS), gas chromatography-MS (GC-MS), and NMR to gain insight into the active chemical compounds present in the plant.

## Materials and methods

### Plant collection, identification, and extraction

Dried *Citrullus colocynthis* (Source origin: Seeds, description of appearance: dark brown, about 8 mm long and 2 mm thick), *Juniperus phoenicea* (Source origin: Seeds, description of appearance: dark green, about 1 mm long and 1 mm thick), and *Anastatica hierochuntica* (Source origin: Whole plant, description of appearance: yellow brown) were purchased from herbalists in Jeddah, Saudi Arabia (S1 Table). The taxonomic identity of each plant was confirmed by a local plant taxonomist (S1 Table). The plants were washed, crushed in liquid nitrogen, and macerated at 10% in dichloromethane:methanol (1:1) overnight at room temperature. Subsequently, plant materials were centrifuged at 13,000 g for 30 min to remove particulates. Extracts were fractionated using solid-phase extraction (SPE) with Bond Elut C2, PPL, C18, or CN-E columns (Agilent Technologies). The columns were conditioned before fractionation with 1 ml of methanol, 5 ml of CHROMASOLVE water, and 1 ml of acidified CHROMASOLVE water (pH 2). Then, 3 ml of the extract was loaded into the column and eluted with 500 μl of a gradually non-polar 10–100% water:methanol gradient (10% step size). The properties of the extracted materials were used to help pre-select specific chemical structures (aliphatic/aromatic) and chemical properties (polar/non-polar) of the potentially active compounds (S2 Table). The fractions were dried under a vacuum (CentriVap Complete, Labconco, Kansas City, MO, USA).

### Cell culture and compound transfer

HeLa cells (parental HeLa cell line, NIH AIDS reagents and reference program) were cultured under typical conditions at 37°C under 5% CO_2_. They were then plated in 384-well clear-bottomed black plates (Greiner bio-one, Germany) at a density of 2,000 cells per well in 25 μl of Dulbecco’s modified Eagle’s medium (DMEM containing GlutaMAX-1; 4.5g/L D-Glucose; Pyruvate; Gibco, Darmstadt, Germany). The cells were incubated for 24 h at 37°C under 5% CO_2_. Natural product SPE compounds were diluted in DMEM, and 100 μl of the diluted compound was transferred to four of the 384 wells (25 μl per well) to achieve a 10-mM final concentration per well. The plates were then incubated at 37°C for 24 h.

### High-content screening imaging and analysis

After 24 h of incubation, the plates were treated with various fluorescent stains and antibodies [26]. From each SPE fraction, we aimed to assay 10 cellular organelles and regulatory proteins. To avoid overlap between fluorescent stains, four different panel stains were used for each sample. Plates were imaged using an ArrayScanTM VTI HCS reader (Cellomics, Thermo Fisher Scientific) with a 10X Zeiss objective lens. The images were analyzed with the Compartmental Analysis BioApplication (Cellomics, Thermo Fisher Scientific) for a minimum of 500 valid objects. Background correction was applied to all the images before being quantified. Panels were as follows: 1) ER, lysosome, and membrane; (2) nucleus, p53, and caspase-9; (3) nucleus, mitochondria, cytochrome C, and NF-κB; (4) nucleus, actin, and tubulin. The cells were stained by permeabilization, blocking, and washing. We used the following HCS reagents: wash buffer I (10X Dulbecco’s PBS), wash buffer II (10X Dulbecco’s PBS with Tween^®^-20), blocking buffer, and permeabilization buffer (Cellomics, HCS reagents, Thermo Fisher Scientific). Stain-specific information and incubation times are presented in S3 Table. Subsequently, all plates were washed three times with wash buffer, sealed and stored at 4°C until further use. Measurements from the reader were averaged, converted to feature scores, clustered, and analyzed using the multiple experiment viewers option employing hierarchical clustering and Pearson’s correlation [27]. A cytological profile of each compound was produced for each of the tested fractions. In total, we selected 21 core cellular features from 12 cellular markers. The difference between the treated and control values for each feature was normalized to score between -1 and 1. The control wells were incubated with only pure DMEM (no fractions). HCS profiling and analysis followed [26].

### Cell loss and cell cycle analysis

HeLa cells were seeded into 384 wells, treated with various concentrations (0, 1.56, 3.12, 6.25, 12.5, 25, 50 μg/ml) of plant fractions (25 μl/well), and maintained under culturing conditions for 24 h or 48 h. Cells were fixed with 3.7% formaldehyde for 15 min, washed twice with Dulbecco’s phosphate-buffered saline (DPBS), and stained with Hoechst 33342 (OG1726671-Thermo Scientific) prepared in DPBS (1 mg/ml) for 10 min in the dark at room temperature. Staining allowed us to quantify the DNA content and to determine cell numbers using the HCS reader (Cellomics, Thermo Fisher Scientific) and the BGRFR 386–23 filter set. Cell loss was calculated as the percentage of optical density (OD) of the treated cells compared with the negative control (untreated cells):

$$\%\;\mathrm{Cell}\;\mathrm{Loss}=\frac{OD\;of\;experimental\;sample\;(treated\;cells)}{OD\;of\;experimental\;sample\;(negative\;control\;cells)}\times 100$$

The Cell Cycle BioApplication (Cellomics, Thermo Fisher Scientific) automatically categorizes each cell’s total nuclear intensity into a single cell-cycle phase. Cells categorized as having DNA~2N, 2N<DNA<4N, or DNA~4N were assigned the respective cell-cycle phases G0/G1, S, or G2/M. Cells categorized as DNA<2N or DNA>4N were respectively considered to be damaged/apoptotic (low nuclear intensity) or clumped/higher ploidy (high nuclear intensity). Cells were treated with 25μg/ml of the appropriate SPE fraction.

### MitoSOX and membrane permeability test

MitoSox Red (M36008, Life Technologies), an indicator of mitochondrial superoxide production, and cell membrane permeability dye (V35123-ThermoScientific) were prepared according to the manufacturer's instructions. Cells were incubated with 5-mM MitoSOX Red and 2-mM cell membrane permeability dye for exactly 20 min at 37°C in 5% CO_2_ in the dark. The resulting labeled cells were washed gently with phosphate buffer saline (PBS) to remove any excess unbound dye. These cells were fixed with 4% formaldehyde for 20 min, washed twice with PBS, and stained with Hoechst33342 stain for 10 min. They were evaluated on the HCS reader using the following filter settings: BGRFR 485–20 for the permeability dye, BGRFR 549–15 for MitoSOX, and BGRFR 386–23 for Hoechst.

### Caspase-9 activity and the p53 assay

Time-dependent studies of caspase-9 and p53 activities were performed in triplicate using the HCS reader. The cells were treated with various concentrations of plant fractions (25 μl/well) for 24 h or 48 h under the previously described culturing conditions. Next, the cells were fixed with 3.7% formaldehyde for 15 min and washed twice with DPBS. The fixed cells were permeabilized with 0.1% Triton X- 100 in PBS for 17 min and washed twice with DPBS. The samples were then blocked for 30 min and incubated with cleaved caspase-9 (ASP315- Thermo Scientific) and p53 antibodies (MA512557-Thermo Scientific) for 1 h. The cells were washed three times with wash buffer II (prepared in water), washed twice with DPBS, and incubated with goat anti-rabbit IgG550 (GAR-DyLight 550–84541) and goat anti-mouse IgG 488 (GAM-DyLight 488–35502) secondary antibodies for 1 h. The cells were rinsed three times with wash buffer II, and nuclei were stained with Hoechst 33258 (OG1726671-Thermo Scientific). Next, the cells were washed twice with DPBS and 25 μl of PBS. The stained cells were visualized and the images were captured using the HCS reader. Primary and secondary antibodies were prepared in blocking buffer. The cell-profiling bioapplication module was used to quantify the fluorescence intensities of each dye: BGRFR 485–20 for P53, BGRFR 549–15 for Caspase-9, and BGRFR 386–23 for Hoechst.

### Histone H2AX phosphorylation

Cell culturing and preparation were performed as described earlier. The cells were treated with various concentrations of plant fractions (25 μl/well) for 6 h and cultured under the conditions described earlier. Next, the cells were fixed with 3.7% formaldehyde for 15 min and washed twice with DPBS. The fixed cells were permeabilized with 0.1% Triton X- 100 in PBS for 15 min and washed twice with DPBS. The samples were then blocked with 2% fetal bovine serum for 15 min and incubated with anti-Histone H2AX polyclonal A (PA184856-Thermo Scientific) for 1 h. The samples were washed three times with wash buffer II (prepared in water) and washed twice with DPBS. The cells were incubated with goat anti-mouse 488 (GAM-DyLight 488 84540) secondary antibodies for 1 h, rinsed three times with wash buffer II, and the nuclei were stained with Hoechst 33342. Finally, the cells were washed twice with DPBS and 25 μl of PBS. The stained cells were visualized and their images were captured using the HCS reader. Primary and secondary antibodies were prepared in blocking buffer. The cell-profiling bioapplication module was used to quantify the fluorescence intensities of each dye: BGRFR 485–20 for H2AX and BGRFR 386–23 for Hoechst.

### HPLC LTQ Orbitrap mass spectrometry analysis

To separate the extracted natural products, we used a C18 (ZORBAX ECLIPS XDB, 5μ, 4.6x250mm) column (Agilent Technologies) with a gradient composed of water/acetonitrile to achieve the highly resolved chromatography. The mobile phase solvents were composed of 100% water + (0.1% formic acid) (A) and 100% acetonitrile + (0.1% formic acid) (B); the injection volume was 10 μL, and the flow rate was set to 450 μL/min. Xcalibur^TM^ software (Thermo Scientific) was used to develop and treat the data.

We used a Thermo LTQ Velos Orbitrap mass spectrometer (Thermo Scientific) equipped with an electrospray ionization source. The mass scan range was set to 100–2000 m/z with a resolving power of 100,000 m/z. The m/z calibration of the LTQ-Orbitrap analyzer was performed in the positive electrospray ionization mode using a solution containing caffeine, MRFA (met-arg-phe-ala) peptide, and Ultramark 1621 according to the manufacturer’s guidelines. We performed this analysis with a heated ion source equipped with a metal needle and operated at 4 kV. The source vaporizer temperature was adjusted to 350°C, the capillary temperature was set to 250°C, and the sheath and auxiliary gases were optimized and set to 40 and 20 arbitrary units, respectively. The bioactive fraction of *J*. *phoenicea* was identified by considering the measured mass and the mass provided by online software, such as Metlin, MetFrag, and Chemspider. To confirm the identity of the identified product, we performed MS/MS studies.

### Chemical analysis by gas chromatography-mass spectrometry

GC-MS was performed on 20% eluted methanol fractions of *J*. *phoenicea* on C2 column cartridges. The setup comprised an Agilent 7890A GC system with split injection (280 C;10:1) coupled with an Agilent MS model 5975C with a triple-axis detector (Agilent Technologies, USA) and a HP-5MS capillary column (30 m X 250 m; film thickness: 0.25 m) (Agilent Technologies, USA). The gas chromatography began with an oven temperature of 50°C for 1 min, which then increased to 300°C for 35 min under constant helium pressure (10 psi). Samples were dissolved in methanol and a 1-μl aliquot was automatically injected. Compounds were identified by matching their EI-MS spectra with those in the National Institute of science and technology (NIST) 2011 Mass Spectral Library using MSD ChemStation (Agilent Technologies).

### Nuclear magnetic resonance analysis

The NMR sample was prepared by dissolving the bioactive fractions in 600 μl of deuterated water (D_2_O,). Then, 550 μl of the solution was transferred to 5-mm NMR tubes. NMR spectra were acquired using a Bruker 600 AVANAC III spectrometer equipped with a multinuclear broadband observe (BBFO) probe (BrukerBioSpin, Rheinstetten, Germany). To achieve a high signal-to-noise ratio, we recorded the ^1^H NMR spectra by collecting 4 k scans with a recycle delay time of 2 s. To suppress the water peak, we induced each spectrum with an excitation sculpting pulse sequence using a standard (zgesgp) program from the Bruker pulse library. The free induction decay (FID) data were collected with a spectral width of 18,028 Hz digitized into 32 k data points, and the FID signals were zero-filled and amplified by an exponential line-broadening factor of 1 Hz before Fourier transformation. Bruker’s Topspin 2.1 software was used in all experiments to collect and analyze the data.

### Statistical analysis

All statistical analyses were performed using GraphPad Prism Version 6 (GraphPad Software, La Jolla, CA, USA). All data are representative of at least four replicates and means ± SD are reported unless otherwise indicated. Statistical significance of a comparison between two groups was determined by a two-tailed Student's t-test where indicated. Significant differences were considered at *p-values* of less than or equal to 0.05.

## Results

### Cytological profiling of natural plant products from Saudi Arabia

**Fig 1:**
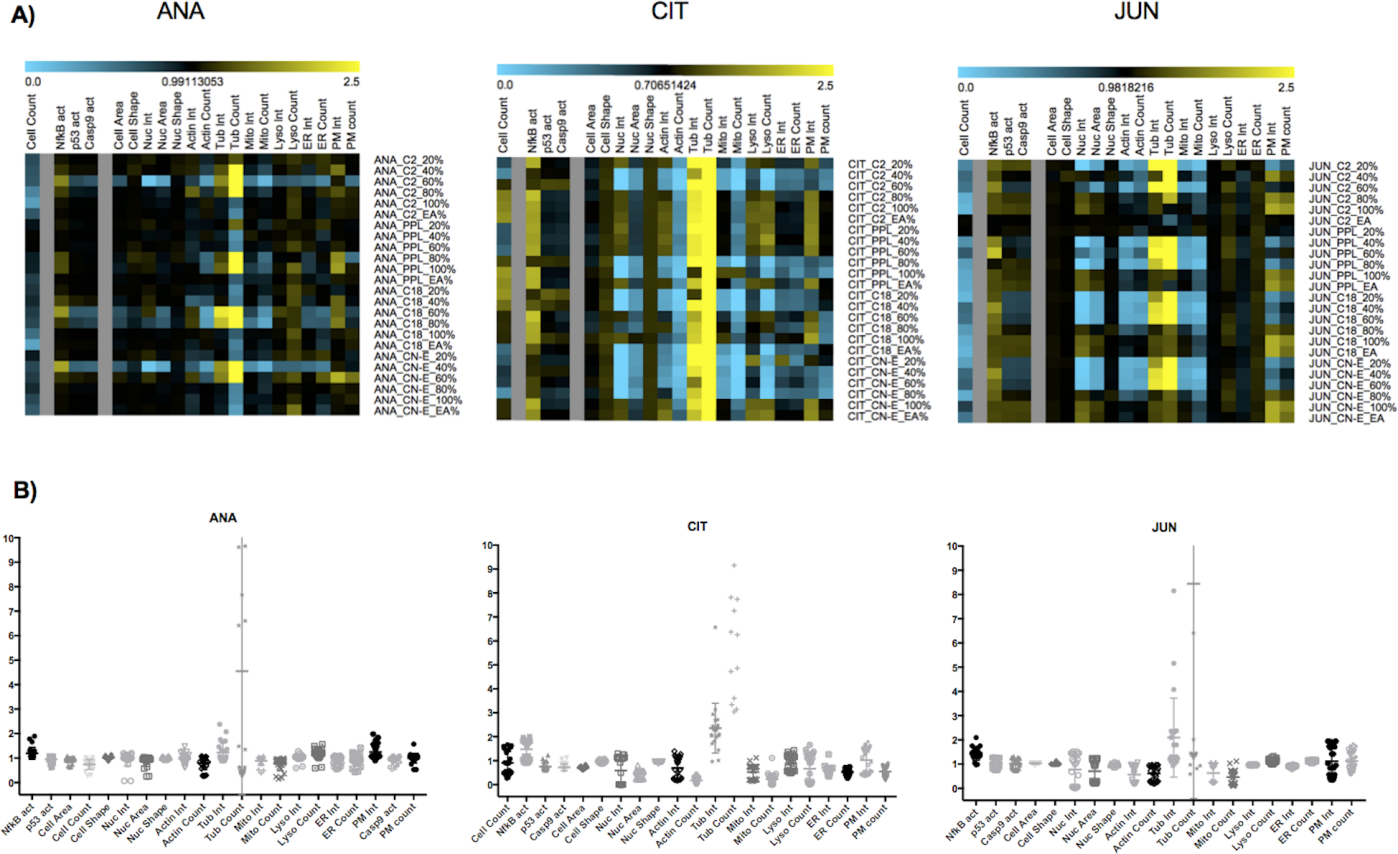
Analysis of core cellular features of HeLa cells treated with plant fractions. A) Heat map data for *A*. *hierochuntica* (ANA), *C*. *colocynthis* (CIT), and *J*. *phoenicea* (JUN) in relation to the core cellular features. Individual plant fractions are presented on the y-axis and individual core cellular features are presented on the x-axis. Positive deviations from HeLa cells treated with plant fractions are displayed in yellow and negative deviations are displayed in blue. B) Summary of the data for each plant with respect to the core cellular features.

In total, 84 natural-product fractions (28 from each of three plants) were obtained in triplicate and screened. Feature scores were calculated in relation to internal controls on each plate. We used a set of core cellular features [26] to reduce the dimensional space defined by a set of factors reflecting the major underlying phenotypic attributes (S4 Table). All three plants showed a positive response to the marker tubulin (Fig 1A and 1B). The heat map profiles of *C*. *colocynthis* (CIT) and *J*. *phoenicea* (JUN) exhibited similar effects on some markers: both had negative effects on mitochondria, actin, nuclear area, and intensity and positive effects on NF-κn. *A*. *hierochuntica* (ANA) was generally active with fractions high in methanol, but cell counts were lower at the higher solvent concentrations found in the C2 and C18 fractions. CIT did not significantly affect cell number whereas JUN significantly reduced cell number in most fractions. ANA fractions eluted from C2 with 60% methanol, from C18 with 60% and 80% methanol, and from CN-E with 40% methanol had negative effects on nuclear intensity and area, mitochondria, actin, and endoplasmic reticulum. CIT fractions had very strong negative effects on lysosomes and slightly negative effects on endoplasmic reticulum and membrane permeability. All active fractions of JUN had minor negative effects on membrane permeability (Fig 1A and 1B).

### Cytological profiling of plant fractions in comparison to reference compounds

**Fig 2:**
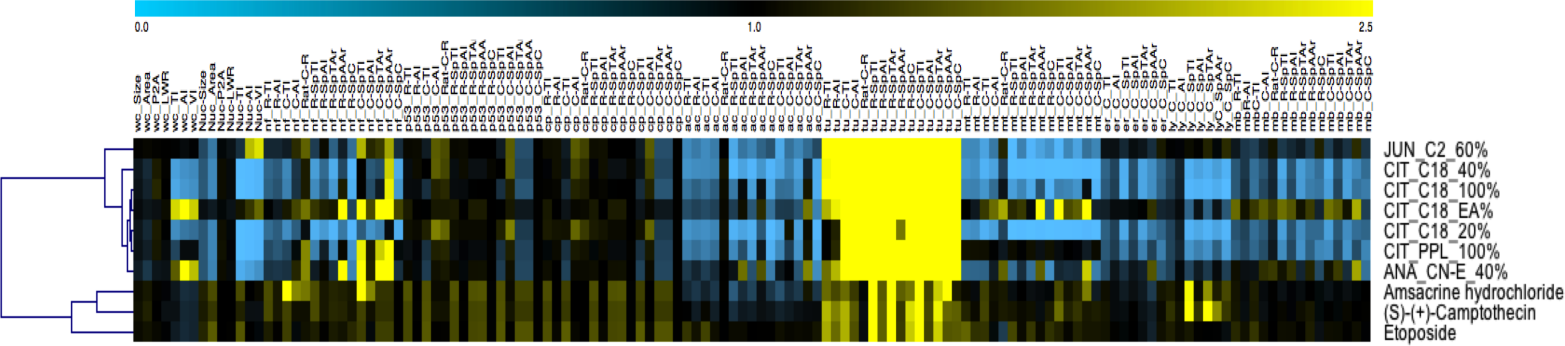
Cytological profiling heat map. Automated HCS was used to assess compound-related perturbations of human cells using a full set of cellular markers. To assign possible biological targets to the test compounds, we compared the resulting cytological profiles to profiles retrieved from reference compounds with known modes of action. Some of the plant fraction extracts closely matched FDA-approved anticancer drugs and clustered with topoisomerase inhibitors. Individual features are presented on the x-axis and individual compounds are presented on the y-axis.

Possible biological targets were identified by a comparison of similarities in high-resolution cytological profiles (consisting of more than 130 cellular features) of the plant fractions to reference compounds with known modes of action. The library of reference compounds contains 735 compounds (LOPAC1280) that affect a variety of known cellular targets, including apoptosis, G proteins and cyclic nucleotides, gene regulation and expression, ion channels, lipid signaling, multi-drug resistance, neurotransmission, and phosphorylation. The reference compound library was screened, stained, and analyzed using the same method applied to the plant fractions [26]. Cluster analysis generated data both from the reference compounds and plant fractions. We found that several fractions closely matched Food and Drug Administration (FDA) -approved anti-cancer drugs, including the topoisomerase inhibitors etoposide, camptothecin and amsacrine hydrochloride (Fig 2).

### Assessment of cell loss and cell cycle arrest

**Fig 3:**
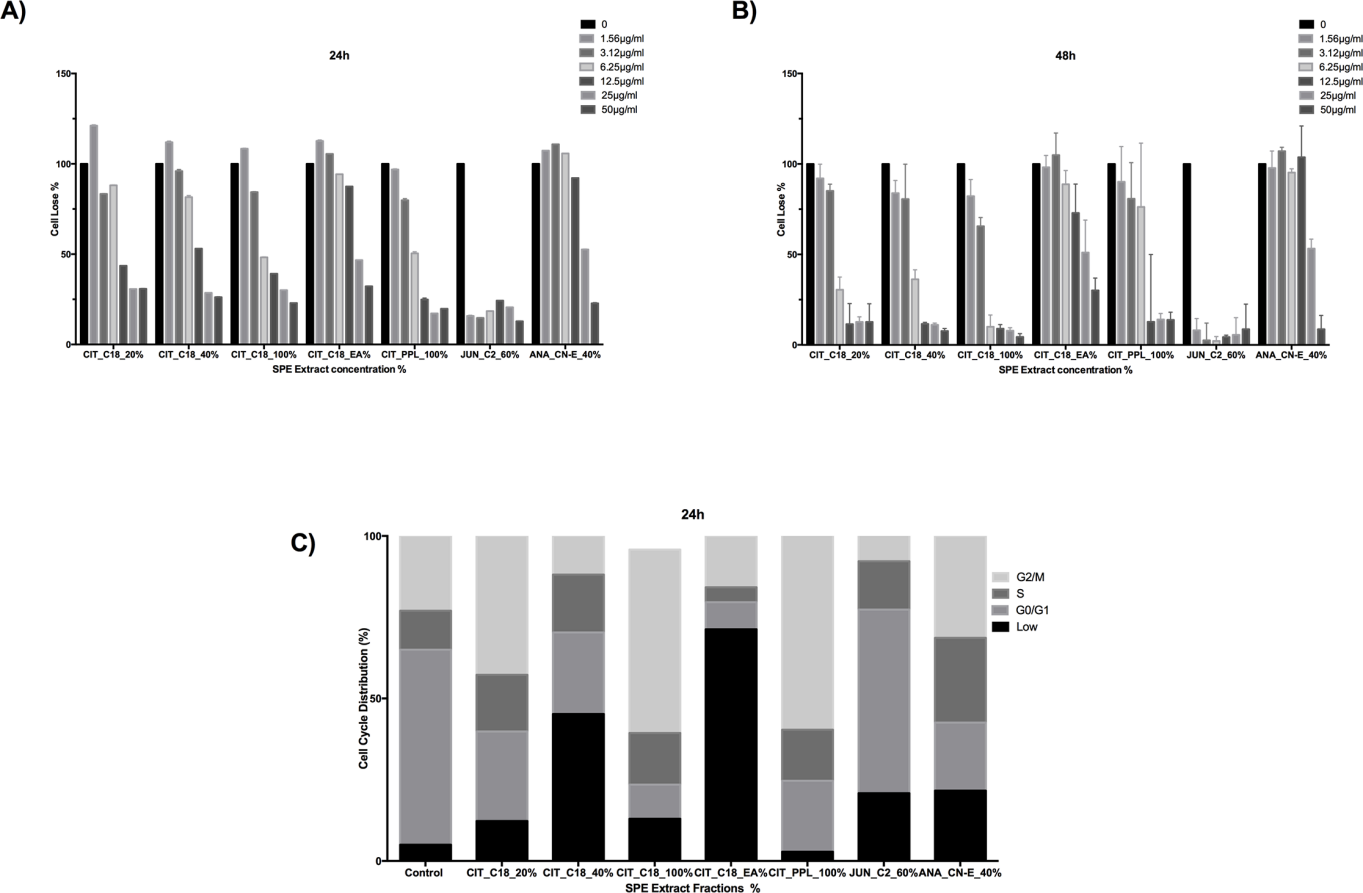
The cytotoxic effect of plant fractions on HeLa cells. A-B) Cells were treated with various concentrations of plant extracts for 24 h or 48 h, stained with Hoechst, and assessed using HCS. The cells exhibited cytotoxicity, which is indicator of induced apoptosis or necrosis. C) The distribution of cells during the cell cycle: G2/M, S, G0/G1 and low phases after a 24-h treatment with plant fractions. Data shown are means ± SD.

Cytotoxic anti-cancer drugs have the potential to elicit cancer cell death by apoptosis or cell necrosis. To evaluate the anti-cancer effect of plant cell fractions extracted by solid-phase extraction (SPE), we incubated HeLa cells with various concentrations (1.56, 3.12, 6.25, 12.5, 25, and 50 μg/ml) of plant cell fractions for 24 h or 48 h. After 24 h of incubation, CIT plant fractions at 12.5 μg/ml concentration caused a loss of HeLa cells whereas ANA_CN-E_40% plant fractions caused a loss of HeLa cells only at higher concentrations (25 and 50 μg/ml). The most prominent decrease in the number of HeLa even at the lowest concentration (1.56 μg/ml) was caused by the JUN_C2_60% fraction (Fig 3A). After 48 h of incubation, with the exception of CIT_C18_EA%, which reduced the number of HeLa cells only at 12.5 μg/ml, all CIT plant fractions caused loss of HeLa cells at 6.25 μg/ml. ANA_CN-E_40% exhibited toxicity only at the highest concentrations (25 and 50 μg/ml), while JUN_C2_60% had the strongest cytotoxicity effect on HeLa cells (Fig 3B).

Next, we investigated whether the plant fractions affected the cell cycle. Our data suggest that several extracted fractions induced cell-cycle arrest at different phases (Fig 3C). Results indicated that plant fractions increased the proportion of Sub-G1 cells, indicating increased numbers of apoptotic cells. Generally, fractions of *C*. *colocynthis* and ANA_CN-E_40% induced cell-cycle arrest in the G0/G1 phase; CIT_C18_EA% induced cell-cycle arrest in the S phase; and JUN_C2_60% induced cell-cycle arrest in the G2/M phase.

### MitoSOX and membrane permeability

**Fig 4:**
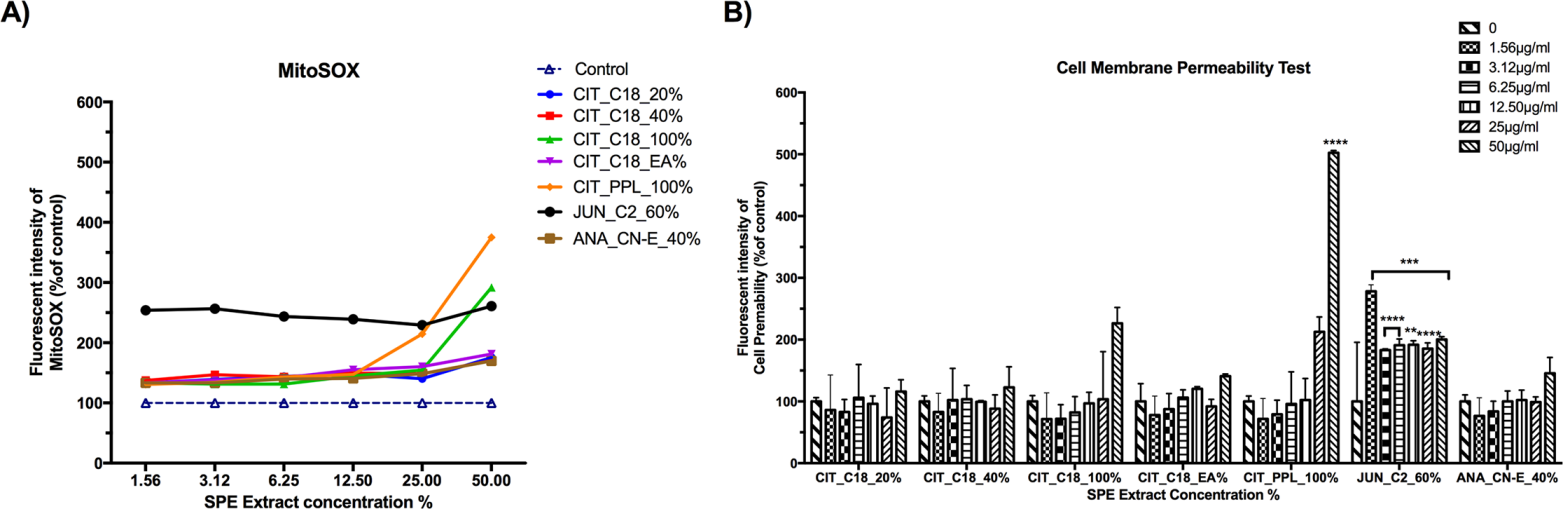
The effect of plant fractions on mitochondrial superoxide production and cell-membrane permeability. **A)** HeLa cells were treated with plant fractions for 24 h. An increase in the MitoSox signal was detected and correlated with the increasing concentration of the plant fraction. **B)** HeLa cells were treated with different concentrations of plant fractions for 24 h and then subjected to a cell membrane permeability test. Fluorescence readouts were normalized against an in-plate control. Each sample was tested in quadruplicate. Data are presented as means ± SD.

To test for production of mitochondrial superoxide, we measured MitoSOX Red fluorescence in the mitochondrial compartment. Mitochondria play a fundamental role in apoptosis, which can be triggered by increased reactive oxygen species (ROS). MitoSOX oxidation was significantly higher in all extracts compared with under control conditions. JUN_C2_60% produced significant amounts of mitochondrial superoxide in a dose-dependent manner (Fig 4A). Fig 4B shows the effect of SPE plant fractions on the membrane permeability of HeLa cells after 24 h: among the CIT fractions, only CIT_PPL_100% caused a significant change in membrane permeability and only at the highest concentration of 50 μg/ml (****p<0.0001); all concentrations of JUN_C2_60% caused a significant change, with the most significant change evident at the lowest concentration of 1.56 μg/ml (***p< 0.0003).

### Characterization of cell apoptosis signaling genes: activation of caspase-9 and p53

**Fig 5:**
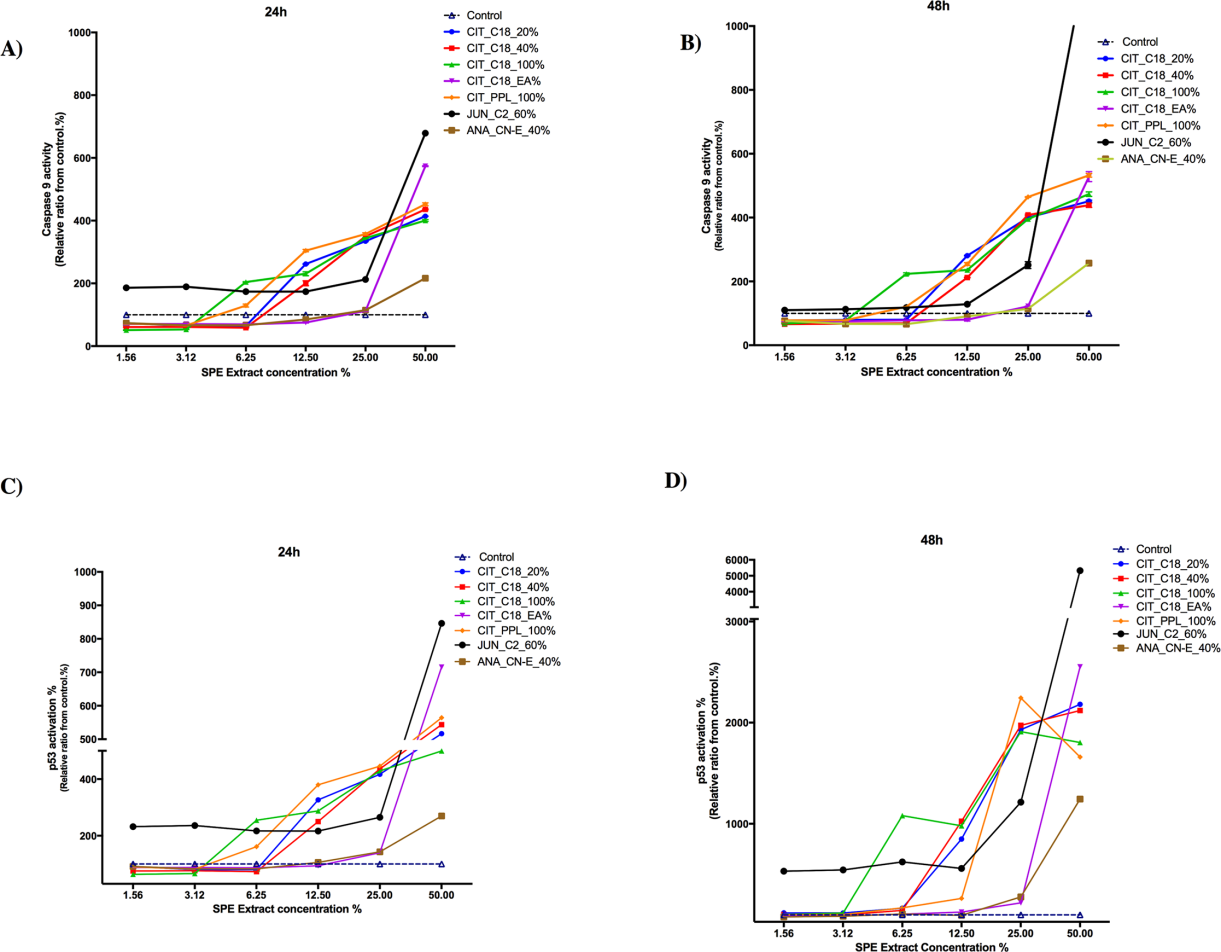
Plant fractions induced an apoptotic effect on HeLa cells. HeLa cells were treated with several concentrations (1.56, 3.12, 6.25, 12.5, 25, and 50 μg/ml) of plant fractions for 24 h or 48 h. A, B) Automated HCS was used to measure the activity of caspase-9 and C, D) p53. The fluorescence readout was normalized against an in-plate control. Each sample was tested in quadruplicate. Data are presented as means ± SD.

To study the molecular mechanism underlying apoptotic processes, we tested cells for the activation of caspase-9 and p53 in a dose- and time-dependent manner. Caspase activation plays a vital role in the initiation and progress of apoptosis. As shown in Fig 5, the activity of caspase-9 increased markedly at 24 h and remained high even after 48 h of treatment, indicating the increasingly toxic effect of the extract on HeLa cells. Treatment with JUN_C2_60% led to the highest activation of both caspase-9 and p53 starting from the lowest concentration (3.12 μg/ml), indicating that the apoptotic signaling pathway had been activated.

### Assessment of the double-strand breaks in DNA from the level of histone H2AX phosphorylation

**Fig 6:**
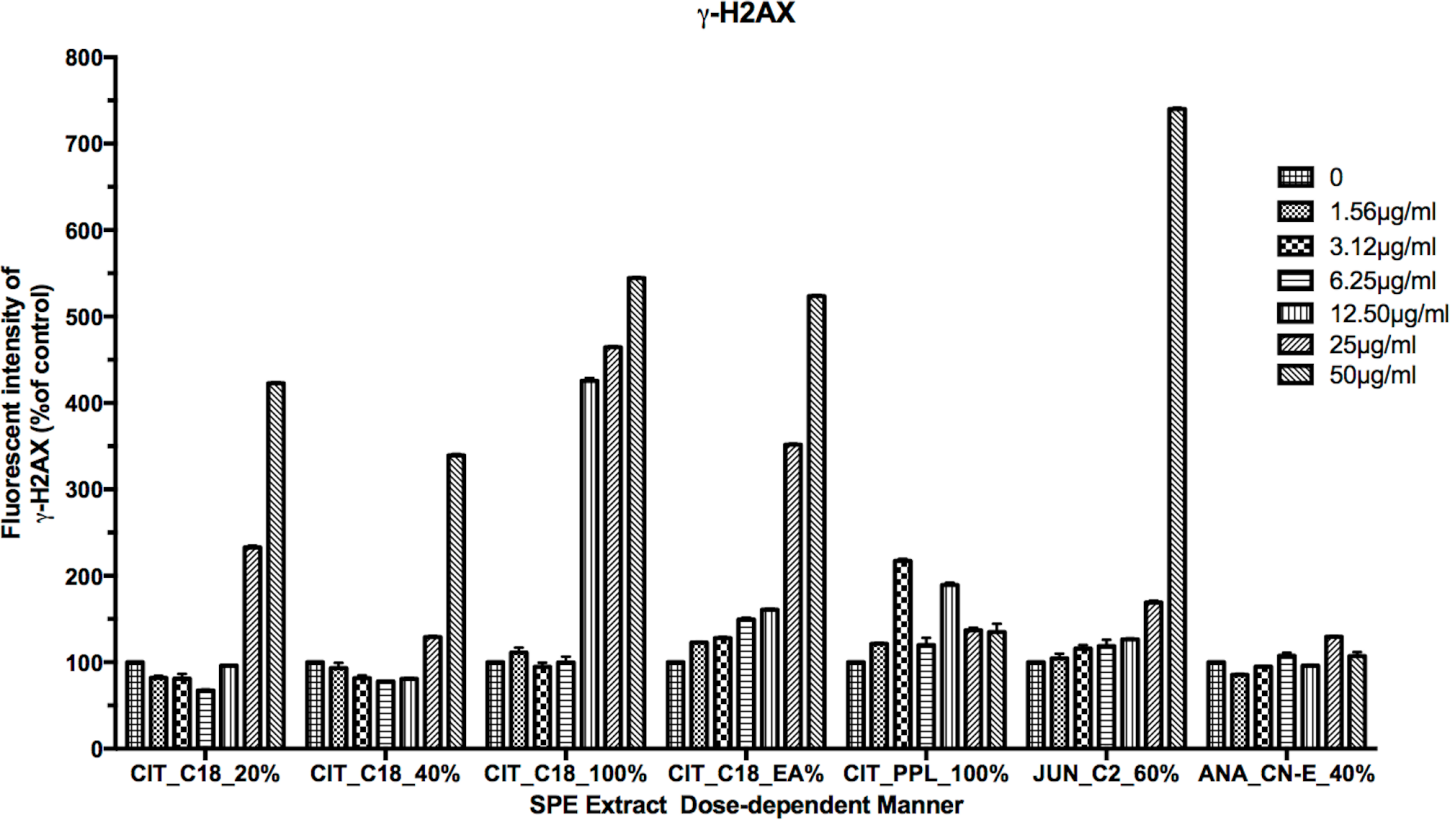
Assesment of the double-strand breaks in the DNA. HeLa cells were treated with different concentrations of SPE fractions to detect the expression of γ-H2AX. Data are presented as means ± SD.

The induction of histone H2AX phosphorylation is a good cell marker of an SPE extract’s genotoxcity. We observed from our HCS results that when incubated with SPE plant fractions, the DNA of HeLa cells exhibit double-strand breaks and histone H2AX is rapidly phosphorylated. As shown in Fig 6, fractions from increasing concentrations of *C*. *colocynthis* induced a distinct accumulation of histone H2AX: CIT_C18_20% and CIT_C18_100% had significantly more histone H2AX than did the control (CIT_C18_20% = 25 μg/ml, **p<0.0002 and CIT_C18_100% = 50 μg/ml, ****p<0.0001). JUN_C2_60% induced significant DNA damage in a different dose-dependent manner: 3.12 μg/ml, 6.25 μg/ml, 25 μg/ml, and 50 μg/ml had p-values of **p< 0.00029, **p< 0.0003, ****p< 0.0001, ****p<0.0001, respectively. Treatment with ANA_CN-E_40% showed no significant induction of histone H2AX.

### Chemical analysis

To identify bioactive compounds in the JUN_C2_60% fraction extract, we subjected samples to extensive chemical analysis using GC-MS, LC-MS, and NMR. GC-MS results showed that the majority of compounds matched the mass spectra of compounds in the NIST library, including 2,2-dimethoxybutane, 2,6-dimethylbenzaldehyde, 3-trifluoroacetoxydodecane, 2,4-ditert-butylphenol, Ethyl 4-ethoxybenzoate, dodecyl acrylate, Stearic acid, hexanedioic acid, bis(2-ethylhexyl) ester and propanoic acid, 3,3'-thiobis-,didodecyl ester (S5 Table).

We identified eight peaks in the LC-MS chromatogram (S1 Fig), which corresponded to the following analytes: (1) ethephon, (2) (+)-eudesmin or (3)burseran and (4)sphinganine, (5) palmitic amide, (6) acesulfame-Na, (7) methyl6-O-[2,3,4-tris-O-(2,2-dimethylpropanoyl)-6-methyl-β-D-glucopyranuronosyl]-βDgalactopyranoside triacetate, and (8) estra-1,3,5(10)-triene-3,6beta,17beta-triol triacetate (S2–S8 Figs). We conducted MS/MS fragmentation studies on some compounds to determine their chemical structures (S12–S14 Figs). These compounds are listed in S6 Table. Next, we used high-resolution NMR spectroscopy to record the proton NMR spectrum of the JUN_C2_60% fraction extract. The ^1^H NMR spectrum exhibited peaks in the aliphatic region, including signals between 0.8–1 ppm, which are generally associated with CH_3_ signals, and a strong peak at 1.13 ppm, confirming that the sample contains many CH_2_ groups (S10 Fig) [28–31]. The broad peak at 1.3 ppm is usually associated with lipid or lipid-like molecules with several adjacent (CH_2_) groups, which supports observations from MS studies for the presence of analytes 4 and 5 [32, 33]. The spectrum also shows broad peaks in the regions of sugar and OH (S10 Fig). Moreover, the ^1^H NMR spectra support MS results that indicate the presence of aromatic molecules, such as analytes 6 and 7, as summarized in S11 Fig.

## Discussion

This study demonstrates the capability of HCS for cytological profiling for drug discovery in natural products. In recent years, HCS has developed from a promising concept into an efficient methodology and indispensable tool. HCS now can be implemented during the early drug discovery process as a result of its recent technological advances [11, 34].

The chemical composition, mode of action, and toxicity of Saudi Arabian plants with medicinal properties have previously not been determined [35]. Here, we use cytological profiling to characterize three native Saudi Arabian plants, *C*. *colocynthis*, *J*. *phoenicea*, and *A*.*hierochuntica*. We used a library of known small molecules with assigned modes of action as reference compounds. We compared their cytological profiles with profiles retrieved from the fractionated plant extracts. We were able to predict the mode of action of pure compounds as well as fractionated extracts [26, 34, 36]. Cluster analysis of the cytological profiles revealed close similarities between seven extracted fractions and topo II inhibitors. Because topoisomerase enzymes are among the primary targets of chemotherapy treatment, we conducted additional experiments to evaluate the effect of these seven fractions on cancer cells. We found that five extracted fractions of *C*. *colocynthis*, a plant found abundantly in Saudi Arabia, were phenotypically similar to the anti-cancer drugs etoposide and camptothecin, which induce topo II formation [37, 38] and activate several molecules, such as histone H2AX, p53, ATM, and Chk1/2, which trigger responses to DNA damage [39, 40].

Our data confirmed that the extracted fractions had cytotoxic effects on HeLa cells, sharply decreasing HeLa cell numbers in a dose- and time-dependent manner (Fig 3A and 3B). We then studied the effect of the extracted fractions on mitochondrial stress, cell membrane permeability, and cell-cycle arrest. Mitochondrial superoxide was detected by MitoSOX Red fluorigenic dye. MitoSOX fluorescence localizes in mitochondria due to its hydrophobic nature and its positively charged triphenylphosphonium moiety [41, 42]. Previous research showed that oxidative stress due to an increased number of reactive oxygen species is a signature of selectivity for tumor toxicity [40, 43]. We found that JUN_C2_60% treatment increased tumor toxicity most significantly and that the permeability caused to the cell membrane led to cell death.

Because cell-cycle dysfunction is a characteristic of cancerous cells, a natural product that is capable of blocking the cell cycle could be considered a potential anti-cancer compound [44]. Our findings showed that fractions of *C*. *colocynthis* and ANA_CN-E_40% caused strong cell-cycle arrest in the G0/G1 phase while JUN_C2_60% caused cell-cycle arrest in the G2/M phase (Fig 3C). Although topoisomerase inhibitors typically exhibit similar cytotoxic affects, they have a different affect on the cell cycle [39].

Topoisomerase forms double-strand breaks in DNA that are necessary for repairs; however, if the strands are not reconnected, cell death can ensue. Therefore, when topo II inhibitor drugs form a complex between topoisomerase and DNA, DNA goes unrepaired, resulting in apoptosis [45–47]. The formation of double-strand breaks during the DNA replication process correlates well with an initial increase in γ-H2AX, which is considered a marker for stalled and collapsed replication forks and reduced topoisomerases I and II activities. Therefore, H2AX levels indicate DNA damage and act as a marker for double-strand break formation [46, 48–51]. In addition, increases in histone H2AX phosphorylation have been observed to correlate with cell-cycle arrest and the activation of apoptotic signaling pathways, such as caspase-9 and p53 [52–54]. We evaluated the abundance of phosphorylated H2AX as an indicator of DNA damage. We found that all treatments with *C*. *colocynthis* and the JUN_C2_60% treatment had significantly high levels of phosphorylation, indicative of DNA damage (Fig 6).

Our results also showed that treatment of HeLa cells with all tested concentrations of *C*. *colocynthis* and JUN_C2_60% caused increased caspase-9 and p53 tumor-suppressor protein activities (Fig 5) Although p53 plays several roles in regulating the cell cycle, its overexpression is associated with obstructing cell growth and inducing apoptosis at the G0/G1 cell cycle checkpoint [55–57]. Moreover, activation of caspase is integral to apoptosis [58]. The upregulation of these two proteins is thus indicative of DNA damage.

Particularly high levels of γH2AX expression in cells with G2/M-phase DNA content suggest that the JUN_C2_60% fraction may be a potential topo inhibitor. Because JUN_C2_60% also contributes to the apoptotic pathway, we subjected it to chemical profiling to find several structures in common with compounds known to have medicinal value.

The 2,2-dimethoxybutane (PubChem CID: 137941) found in the JUN_C2_60% fraction was found to be toxic to microbial membranes [59–61], but it has heretofore not been identified as an anti-cancer compound. 2,4-ditert-butylphenol is a phenolic compound and a secondary metabolite in plants. It has been reported to exhibit strong antioxidant activity, anti-cancer activity, antifungal activity and antibacterial activity [59, 62–64]. It is cytotoxic to MCF7 cells (IC50 value of 5.75 μg/mL), KB cells (IC50 value of 0.81 μg/mL) and CasKi cells (IC50 value of 4.5 μg/mL). Dharni *et al*. studied the antifungal effect of this compound and concluded that it potentially binds to β-tubulin in microtubules, inhibiting eukaryotic cell growth by destroying their dynamic instability while affecting cytoskeletal polymers [64]. This may suggest that the JUN_C_2__60% fraction could affect β-tubulin. Our HCS panel reveals clear effects on β-tubulin, but further studies using different fraction concentrations to clarify the exact effects on β-tubulin microtubules are needed (Fig 4). 3-Trifluoroacetoxydodecane (PubChem CID: 534402) has been reported to have anti-cancer and antimicrobial activities [65]. Stearic acid (PubChem CID: 5281), a saturated long-chain fatty acid with an 18-carbon backbone, is found in various animal and plant fats. It is a major component of cocoa butter and shea butter. Stearic acid has been reported to exert anti-cancer as well as anti-inflammatory effects [66, 67]. It has been reported that different concentrations of stearic acid inhibited growth of human cervical cancer cells (HOG-1) and DNA synthesis. It has also been shown to affect early cell proliferation signals [68], in agreement with our finding that *J*.*phoenicea* extract inhibits cell growth in the G2/M phase and leads to high levels of γH2AX expression, which is a marker of DNA damage. Stearic acid was previously found in *J*. *phoenicea* from Southern Tunisia [69]. Anti-inflammatory activities have been attributed to propanoic acid, 3,3'-thiobis-,didodecyl ester (PubChem CID: 31250) [70]. Ethyl 4-ethoxybenzoate (PubChem CID: 90232) has been reported to be a local anesthetic [71]. Of the detected compounds, dodecyl acrylate (PubChem CID: 75084), 2,6-Dimethylbenzaldehyde (PubChem CID: 583841) and hexanedioic acid,bis(2-ethylhexyl) ester (PubChem CID: 7641) have not yet been reported to have anti-cancer activity unlike ethephon (PubChem CID: 27982), (+)-eudesmin (PubChem CID: 73117) or burseran (PubChem CID: 11101102) and sphinganine (PubChem CID: 91486) [72–75]. Sphinganine has been reported to be an anti-cancer compound (80). Our results show that sphinganine is associated with the activation of p38 MAPK and JNK and with the weak inhibition of AKT, which explains the DNA damage and activation of p53 by a *J*. *phoenicea* fraction. Our LC-MS results revealed the presence of Methyl6-O-[2,3,4-tris-O-(2,2-dimethylpropanoyl)-6-methyl-β-D-glucopyranuronosyl]-βDgalactopyranoside triacetate and Estra-1,3,5(10)-triene-3,6beta,17beta-triol triacetate. Estra-1,3,5(10)-triene-3,6beta,17beta-triol triacetate (PubChem CID: 5756) is used as anti-inflammatory drug but no anti-inflammatory activity has been reported for Methyl6-O-[2,3,4-tris-O-(2,2-dimethylpropanoyl)-6-methyl-β-D-glucopyranuronosyl]-β-Dgalactopyranoside triacetate compound.

Our results demonstrate the power of HCS for drug discovery. HCS allows traditionally used medicinal plants to be assessed for the presence of bioactive compounds. Cytological profiles allow us to identify topoisomerase II inhibitor activity in the plant fractions that we tested to verify the presence of anti-cancer compounds in the plants. Further studies are needed to evaluate and better characterize the active compounds identified in our study. This can be done by guided fractionation using HPLC to isolate and eliminate the active from the non-active compounds and to retest the active compounds with HCS. Furthermore, active compounds can be synthesized and tested in vivo.

## Supporting information

S1 Table Summery illustrate information about used plants in this study. (DOCX)

S2 Table Full description of Solid phase extraction cartridges (SPE-Cartridges) for extraction of plants natural products. This figure were adapted from the cited publication.
(DOCX)

S3 Table The panel’s description for the cellular features measured in cytological profiling. (DOCX)

S4 Table The core cellular features markers with parameters measurements and phenotypic attributes are shown in this table. (DOCX)

S5 Table Phytochemicals identified in extract JUN_C2_60% by GC/MS. (DOCX)

S6 Table Proposed identified chemicals from JUN_C2_60% by LC/MS. (DOCX)

S1 Fig LC-MS chromatogram of the studied sample superimposed on an acetonitrile blank. Eight peaks were identified from the LC-MS chromatogram: (1) 185.98322 m/z, (2) 144.98225 m/z, (3) 144.98225 m/z, (4) 288.28995 m/z, (5) 256.26364 m/z, (6) 387.18086 m/z, (7) 415.21219 m/z, and (8) 637.30585 m/z.
(TIFF)

S2 Fig Deconvolution of the ion at 185.98322 m/z; +ve ESI of analyte 1. Acesulfame-K, Chemical Formula: C_4_H_5_NO_4_S.
(TIFF)

S3 Fig Deconvolution of the ion at 144.98158 m/z; +ve ESI of analytes 2 & 3. Ethephon, Chemical Formula: C_2_H_6_ClO_3_P.
(TIFF)

S4 Fig Deconvolution of the ion at 288.29000 m/z; +ve ESI of analyte 4. Sphinganine, Chemical Formula: C17H37NO2.
(TIFF)

S5 Fig Deconvolution of the ion at 256.26355 m/z; +ve ESI of analyte 5. Palmitic amide, Chemical Formula: C_16_H_33_NO.
(TIFF)

S6 Fig Deconvolution of the ion at 387.18086 m/a; +ve ESI of analyte 6. Burseran or (+)Eudesmin, Chemical Formula: C_22_H_26_O_6_.
(TIFF)

S7 Fig Deconvolution of the ion at 415.21219 m/z; +ve ESI of analyte 7. Estra-1,3,5(10)-triene-3,6beta,17beta-triol triacetate. Chemical Formula: C_24_H_30_O_6_.
(TIFF)

S8 Fig Deconvolution of the ion at 637.30585 m/z; +ve ESI of analyte 8. Methyl 6-O-[2,3,4-tris-O-(2,2-dimethylpropanoyl)-6-methyl-β-D-glucopyranuronosyl]-β-D-galactopyranoside. Chemical Formula: C_29_H_48_O_15_.
(TIFF)

S9 Fig Extended CH2 and CH3 regions of the NMR spectrum. The spectrum was recorded at room temperature using a 600-MHz NMR spectrometer.
(TIFF)

S10 Fig Extended OH and sugar regions of the NMR spectrum. The spectrum was recorded at room temperature using a 600-MHz NMR spectrometer. This figure verifies the MS finding of the (Methyl 6-O-[2,3,4-tris-O-(2,2-dimethylpropanoyl)-6-methyl-β-D-glucopyranuronosyl]-β-D-galactopyranoside) molecules with signals of several CH3 groups around 1 ppm in Fig 2.
(TIFF)

S11 Fig This figure verifies the MS finding of the extended aromatic region of the NMR spectrum. A) Estra-1,3,5(10)-triene-3,6beta,17beta-triol triacetate B) Burseran C) (+)Eudesmin D) Extended aromatic region of the NMR spectrum.
(TIFF)

S12 Fig The proposed MS/MSn (n = 2) fragmentation pattern of the identified compound (C_17_H_38_NO_2_) at *m/z* 288.28970. (TIFF)

S13 Fig The proposed MS/MSn (n = 2) fragmentation pattern of the identified compound (C_24_H_31_O_6_) at *m/z* 415.21151. (TIFF)

S14 Fig The proposed MS/MSn (n = 5) fragmentation pattern of the identified compound (C_28_H_48_O_15_) at *m/z* 637.30659. (TIFF)
