# Supplementary material for: Anti-cancer agents in Saudi Arabian herbals revealed by automated high-content imaging
Source: PLoS One. 2017 Jun 13;12(6):e0177316. doi: 10.1371/journal.pone.0177316 (PMC5469452; doi:10.1371/journal.pone.0177316)
Supplement: S1 Table — (DOCX) [file pone.0177316.s001.docx]

Supplementary Table 1: Summery illustrate information about used plants in this study

| **Scientific name** | **Family name** | **Traditional name** | **Code** | **Name and location of the purchase locations** | **Picture** |
| --- | --- | --- | --- | --- | --- |
| *Citrullus colocynthis (L.) Schrad.* | Cucurbitaceae | Hanzal | CIT | Shatie Market, Ahmad Al Attas, Al-Zahra'a, Jeddah 23425, Saudi Arabia | 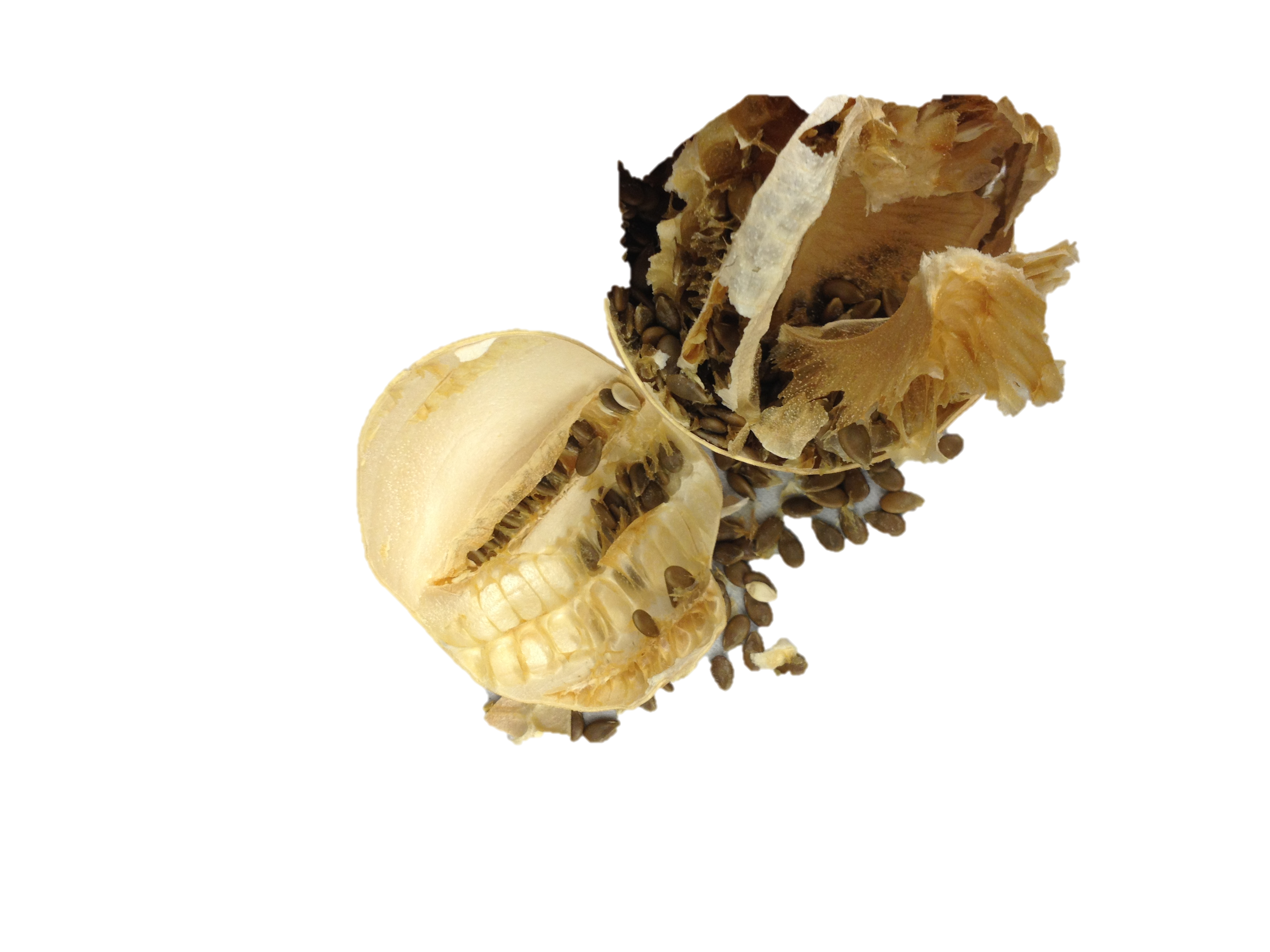 |
| *Anastatica hierochuntia L.* | Brassicaceae | Kaff Maryam | ANA | Etarat Al-hejaz, Jeddah 23436, Saudi Arabia | 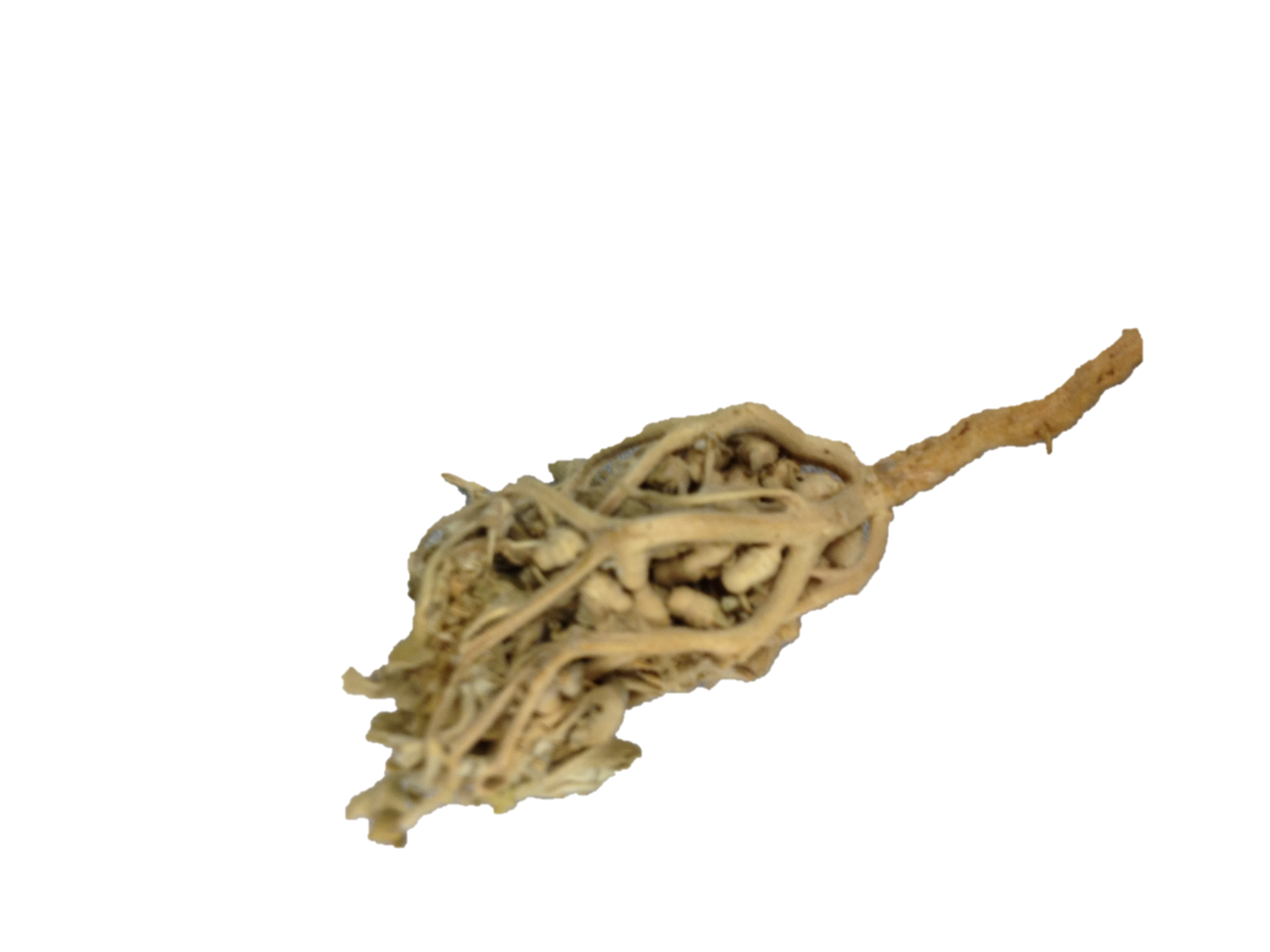 |
| *Juniperus phoenicea Pall.* | Cupressaceae | Arar | JUN | Shatie Market, Ahmad Al Attas, Al-Zahra'a, Jeddah 23425, Saudi Arabia | 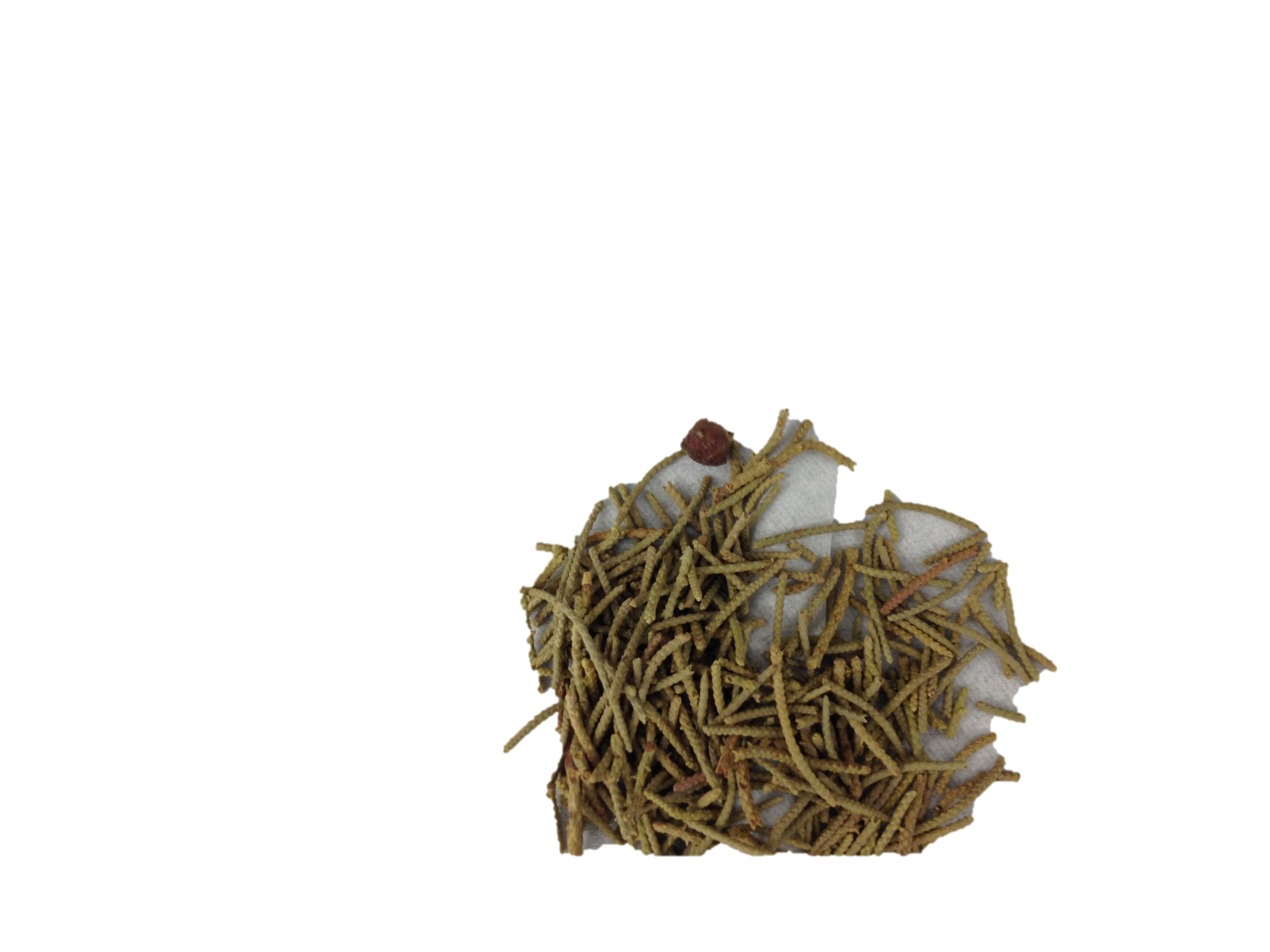 |
