# Supplementary material for: Anti-cancer agents in Saudi Arabian herbals revealed by automated high-content imaging
Source: PLoS One. 2017 Jun 13;12(6):e0177316. doi: 10.1371/journal.pone.0177316 (PMC5469452; doi:10.1371/journal.pone.0177316)
Supplement: S2 Table — This figure were adapted from the cited publication. (DOCX) [file pone.0177316.s002.docx]

**Supplementary Table 2:** Full description of Solid phase extraction cartridges (SPE-Cartridges) for extraction of plants natural products. This figure were adapted from the cited publication[1]

| **Bond Elut SPE-Cartridges** | **Type of Material** | **Properties** | **Primary Retention Mechanism** | **Typical Sample Types** |
| --- | --- | --- | --- | --- |
| C_2_ | Silica based, ethyl bonded, endcapped | Alternative sorbent, if analytes are retained too strongly on C_8_ or C_18_ phases | Weakly nonpolar | Plasma, urine, aqueous samples |
| C_18_ | Silica based ,trifunctional octadecyl bonded,endcapped | Extreme retentive nature for nonpolar compounds, applicable for desalting aqueous matrices | Strongly nonpolar | Water, aqueous biological fluids |
| CN-E | Silica based, cyanopropyl bonded,endcapped | Different selectivity to alkyl and aliphatic functionalized phases due to electron density of the aromatic ring | Moderately nonpolar (aqueous matrix) or polar (nonpolar organic matrix) | Aqueous samples (nonpolar),organic samples (polar) |
| PPL | Styrene-divinylbenzene(SDVB) polymer with a proprietary derivitized nonpolar surface | Extreme hydrophobicity and surface area, achieves high recovery levels and fast extraction speeds | Highly polar | Waste water (phenols) |

1. Jha, B., et al., *Quorum sensing inhibition by Asparagopsis taxiformis, a marine macro alga: separation of the compound that interrupts bacterial communication.* Marine drugs, 2013. **11**(1): p. 253-265.
