# Supplementary material for: Anti-cancer agents in Saudi Arabian herbals revealed by automated high-content imaging
Source: PLoS One. 2017 Jun 13;12(6):e0177316. doi: 10.1371/journal.pone.0177316 (PMC5469452; doi:10.1371/journal.pone.0177316)
Supplement: S3 Table — (DOCX) [file pone.0177316.s003.docx]

Supplementary Table 3: The panel’s description for the cellular features measured in cytological profiling.

| **Panels** | **Cellular feature** | **Fluorescent stain** | **Secondary Antibody** | **Incubation Time** |
| --- | --- | --- | --- | --- |
| Panel 1 | Nucleus | Hoechst Stain (OG1726671-Thermo Scientific) | _____ | 10 min |
|  | ER | ER tracker |  | 30 min |
|  | Lysosome | Lyso tracker |  | 30 min |
| Panel 2 | Nucleus | Hoechst Stain (OG1726671-Thermo Scientific) | GAR550(OC183252)-GAR488(OC183252) | 10 min |
|  |  |  |  | 1 hour |
|  | p53 | P53 (MA512557-Thermo Scientific) |  |  |
|  | Caspase 9 | Cleaved Caspase-9 antibody  (ASP315- Thermo Scientific) |  |  |
|  |  |  |  | 1 hour |
| Panel 3 | Nucleus | Hoechst Stain (OG1726671-Thermo Scientific) | GAR488(OI189170)-GAM650 | 10 min |
|  | Mitochondria | MitoTracker Orange CMTMRos (M7510, life technologies) |  | 30 min |
|  | NFkB | NFkappaB/p65 Antibody  (PA5-16545) |  | 1 hour |
|  | Cytochrome C | Cytochrome C Antibody (MA5-11823-Thermo Scientific) |  | 1 hour |
| Panel 4 | Nucleus | Hoechst Stain (OG1726671-Thermo Scientific) | GAM550 (NJ172004) | 10 min |
|  | Actin | Phalloidin-FITC |  | 1 hour |
|  | Tubulin | Beta-3 Tubulin Antibody (MA1-19187) |  | 1 hour |
