# Supplementary material for: Anti-cancer agents in Saudi Arabian herbals revealed by automated high-content imaging
Source: PLoS One. 2017 Jun 13;12(6):e0177316. doi: 10.1371/journal.pone.0177316 (PMC5469452; doi:10.1371/journal.pone.0177316)
Supplement: S4 Table — (DOCX) [file pone.0177316.s004.docx]

Supplementary Table 4: The core cellular features markers with parameters measurements and phenotypic attributes are shown in this table.

| **Core Cellular Markers** | **Cytological Features** | **Phenotypic attributes** |
| --- | --- | --- |
| Cell count | Valid cell | Cell viability |
| NF-κB act | Circ-ring ratio | Nuclear and cytoplasmic area ratio |
| P53 act | Circ total intensity | Nuclear area total intensity |
| Casp9 act | Circ total intensity | Nuclear area total intensity |
| Cell Area and shape | whole cell marker Area and LWR shape | Cell morphology and cell ellipticity |
| Nuc Int, Nuc Area and Nuc shape | Nuclear Total intensity, nuclear area and nuclear LWR shape | Nuclear size, morphology and ellipticity |
| Actin Int | Ring total intensity | The total intensity of the area around the nucleolus |
| Tub Int and Tub count | Ring total intensity | The total intensity of the area around the nucleolus and count |
| Mito Int and Mito Count | Mitochondria ring total intensity and ring spot count | The intensity of the area around the nucleolus and count |
| Lyso Int and Lyso count | Lysosome Ring total intensity and ring spot count | The intensity of the area around the nucleolus and count |
| ER Int and ER count | Endoplasmic Reticulum Ring total intensity and ring spot count | The intensity of the area around the nucleolus and count |
| PM Int and PM count | Permeability membrane ring total intensity and ring spot count | The intensity of the area around the nucleolus and count |
