# Supplementary material for: Anti-cancer agents in Saudi Arabian herbals revealed by automated high-content imaging
Source: PLoS One. 2017 Jun 13;12(6):e0177316. doi: 10.1371/journal.pone.0177316 (PMC5469452; doi:10.1371/journal.pone.0177316)
Supplement: S5 Table — (DOCX) [file pone.0177316.s005.docx]

**Supplementary Table 5:** Phytochemicals identified in extract JUN_C2_60% by GC/MS

| **S.No.** | **R.Time (min)** | **Proposed Name** | **MF** | **Molecular Formula** | **Molecular Weight** |
| --- | --- | --- | --- | --- | --- |
| 1 | 4.632 | 2,2-Dimethoxybutane | 795 | C_6_H_14_O_2_ | 118 |
| 2 | 12.170 | 2,6-Dimethylbenzaldehyde | 750 | C_9_H_10_O | 134 |
| 3 | 15.461 | 3-Trifluoroacetoxydodecane | 709 | C_14_H_25_F_3_O_2_ | 282 |
| 4 | 15.904 | Phenol, 2,4-bis(1,1 dimethylethyl)- | 862 | C_14_H_22_O | 206 |
| 5 | 16.186 | Benzoic acid, 4-ethoxy-, ethyl ester | 648 | C_11_H_14_O_3_ | 194 |
| 6 | 18.059 | Dodecyl acrylate | 859 | C_15_H_28_O_2_ | 240 |
| 7 | 22.743 | Octadecanoic acid | 631 | C_18_H_36_O_2_ | 284 |
| 8 | 24.731 | Hexanedioic acid, bis(2-ethylhexyl) ester | 697 | C_22_H_42_O_4_ | 370 |
| 9 | 59.772 | Propanoic acid, 3,3'-thiobis-, didodecyl ester | 637 | C_30_H_58_O_4_S | 514 |
