# Supplementary material for: Anti-cancer agents in Saudi Arabian herbals revealed by automated high-content imaging
Source: PLoS One. 2017 Jun 13;12(6):e0177316. doi: 10.1371/journal.pone.0177316 (PMC5469452; doi:10.1371/journal.pone.0177316)
Supplement: S6 Table — (DOCX) [file pone.0177316.s006.docx]

**Supplementary Table 6:** Proposed identified chemicals from JUN_C2_60% by LC/MS.

| **Chromatogram Peaks** | **Molecular Ion** | **Accurate Masse (Δm ≤ 5 ppm)** | **Proposed Name** | **Chemical Structure** | **Software** | **MS/MS (n)** |
| --- | --- | --- | --- | --- | --- | --- |
| **1** | [M_1_+Na]^+^ | 185.98322 | Acesulfame-Na | C_4_H_5_NO_4_SNa | Metlin | NA |
| **2 and 3** | [M_2/3_+H]^+^ | 144.98158 | Ethephon | C_2_H_6_ClO_3_P | Metlin | NA |
| **4** | [M_4_+H]^+^ | 288.29000 | C17 Sphinganine (heptadecasphinganine) | C_17_H_37_NO_2_ | Metlin | MS/MS (2) |
| **5** | [M_5_+H]^+^ | *256.26355* | Palmitic amide | C_16_H_33_NO | Metlin | NA |
| **6** | [M_6_+H]^+^ | 387.18086 | Burseran | C_22_H_26_O_6_ | Metlin | NA |
|  |  |  | (+)Eudesmin | C_22_H_26_O_6_ | Metlin | NA |
| **7** | [M_7_+H]^+^ | 415.21219 | Estra-1,3,5(10)-triene-3,6beta,17beta-triol triacetate | C_24_H_30_O_6_ | Metlin | MS/MS (2) |
| **8** | [M_8_+H]^+^ | 637.30585 | Methyl 6-O-[2,3,4-tris-O-(2,2-dimethylpropanoyl)-6-methyl-β-D-glucopyranuronosyl]-β-D-galactopyranoside | C_29_H_48_O_15_ | Met-Frag | MS/MS (5) |
