# Supplementary figures and images for: Anti-cancer agents in Saudi Arabian herbals revealed by automated high-content imaging
Source: PLoS One. 2017 Jun 13;12(6):e0177316. doi: 10.1371/journal.pone.0177316 (PMC5469452; doi:10.1371/journal.pone.0177316)

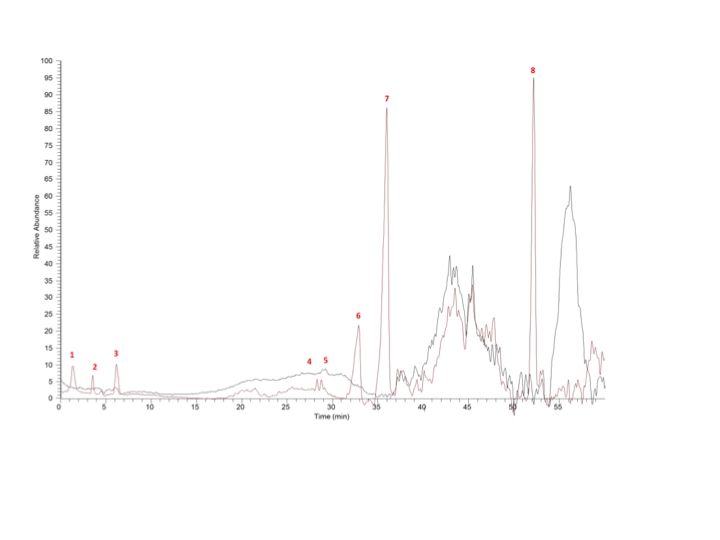

Supplement: S1 Fig — Eight peaks were identified from the LC-MS chromatogram: (1) 185.98322 m/z, (2) 144.98225 m/z, (3) 144.98225 m/z, (4) 288.28995 m/z, (5) 256.26364 m/z, (6) 387.18086 m/z, (7) 415.21219 m/z, and (8) 637.30585 m/z. (TIFF) [file pone.0177316.s007.tiff]

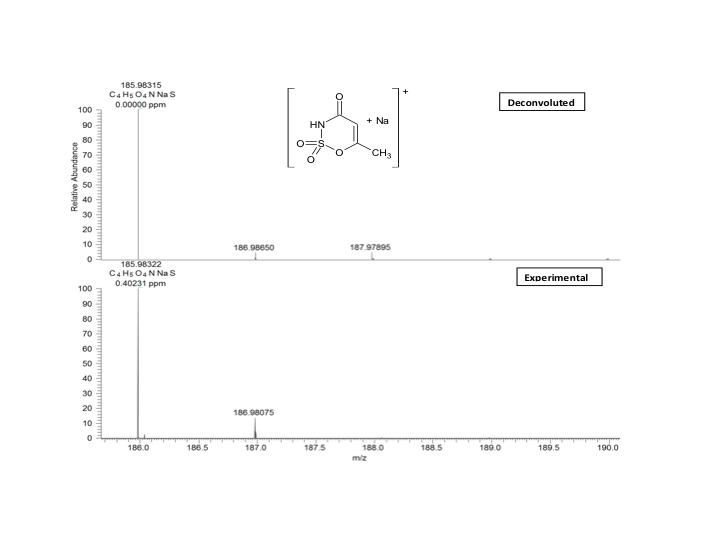

Supplement: S2 Fig — Acesulfame-K, Chemical Formula: C4H5NO4S. (TIFF) [file pone.0177316.s008.tiff]

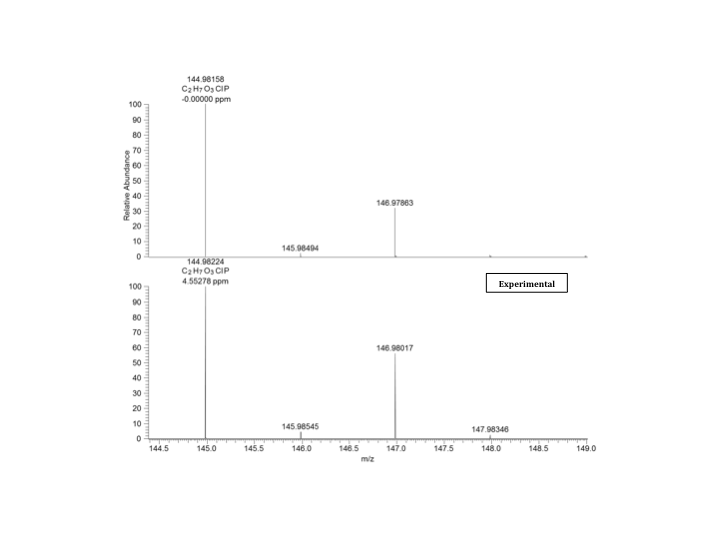

Supplement: S3 Fig — Ethephon, Chemical Formula: C2H6ClO3P. (TIFF) [file pone.0177316.s009.tiff]

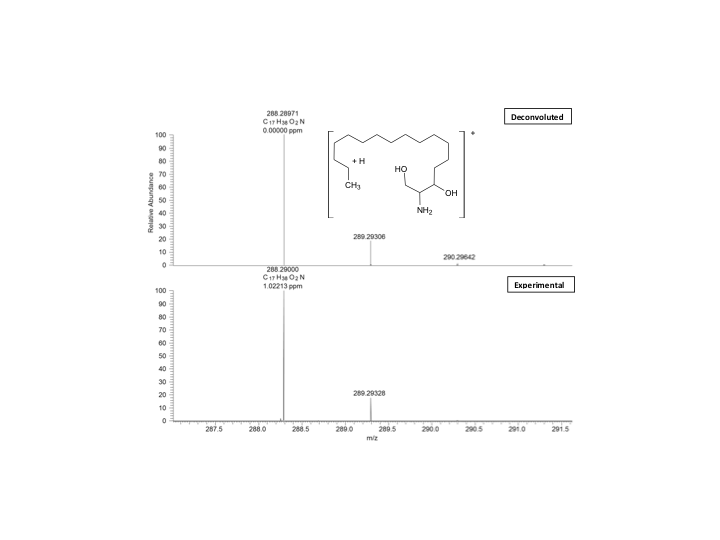

Supplement: S4 Fig — Sphinganine, Chemical Formula: C17H37NO2. (TIFF) [file pone.0177316.s010.tiff]

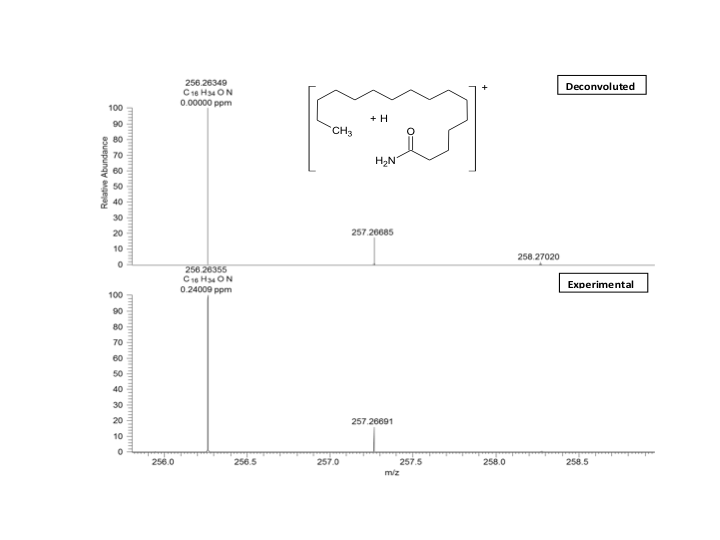

Supplement: S5 Fig — Palmitic amide, Chemical Formula: C16H33NO. (TIFF) [file pone.0177316.s011.tiff]

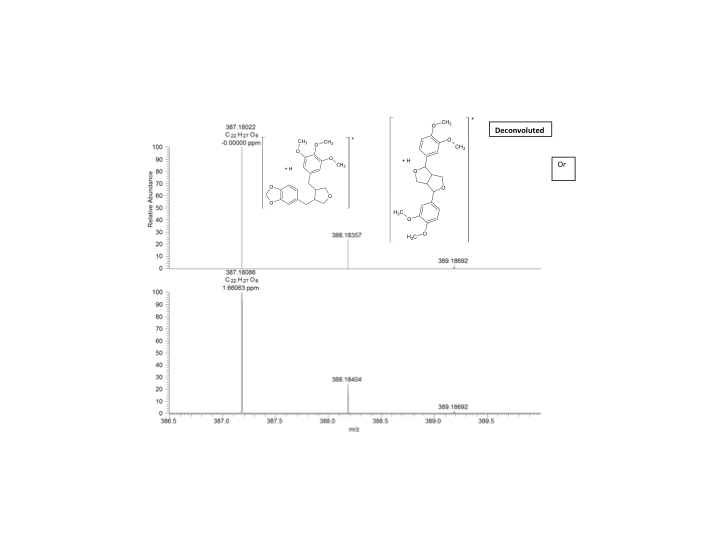

Supplement: S6 Fig — Burseran or (+)Eudesmin, Chemical Formula: C22H26O6. (TIFF) [file pone.0177316.s012.tiff]

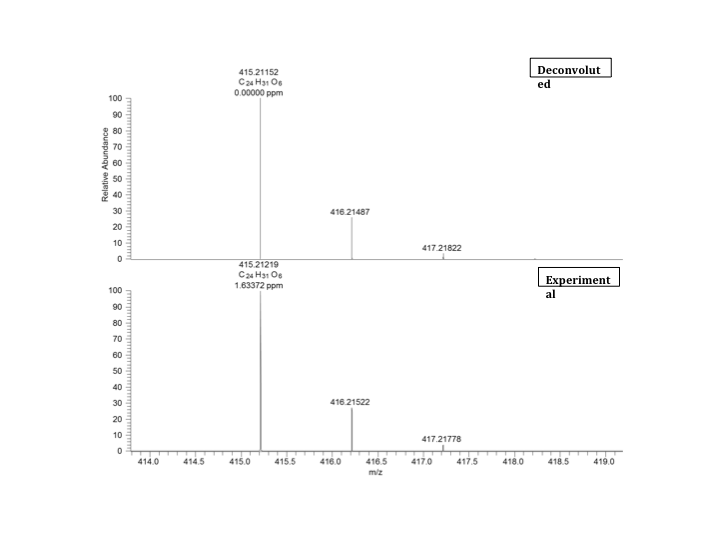

Supplement: S7 Fig — Estra-1,3,5(10)-triene-3,6beta,17beta-triol triacetate. Chemical Formula: C24H30O6. (TIFF) [file pone.0177316.s013.tiff]

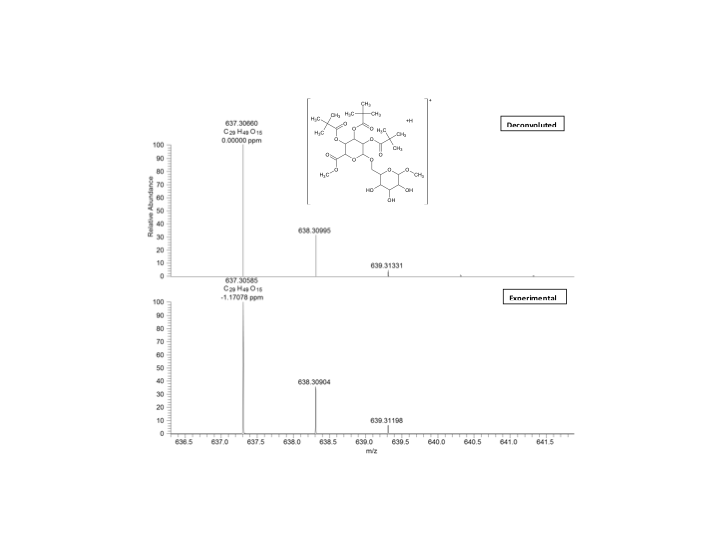

Supplement: S8 Fig — Methyl 6-O-[2,3,4-tris-O-(2,2-dimethylpropanoyl)-6-methyl-β-D-glucopyranuronosyl]-β-D-galactopyranoside. Chemical Formula: C29H48O15. (TIFF) [file pone.0177316.s014.tiff]

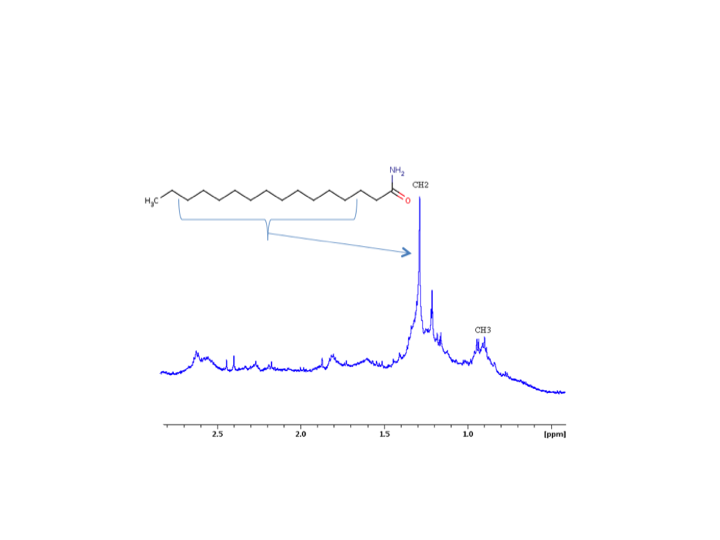

Supplement: S9 Fig — The spectrum was recorded at room temperature using a 600-MHz NMR spectrometer. (TIFF) [file pone.0177316.s015.tiff]

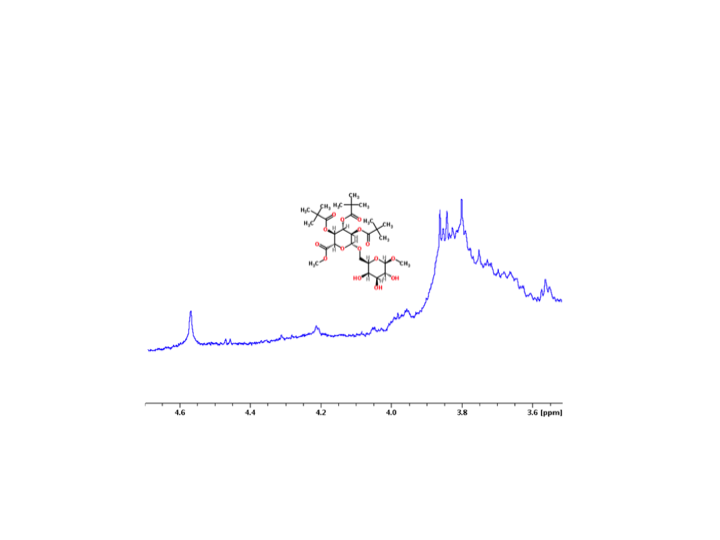

Supplement: S10 Fig — The spectrum was recorded at room temperature using a 600-MHz NMR spectrometer. This figure verifies the MS finding of the (Methyl 6-O-[2,3,4-tris-O-(2,2-dimethylpropanoyl)-6-methyl-β-D-glucopyranuronosyl]-β-D-galactopyranoside) molecules with signals of several CH3 groups around 1 ppm in Fig 2. (TIFF) [file pone.0177316.s016.tiff]

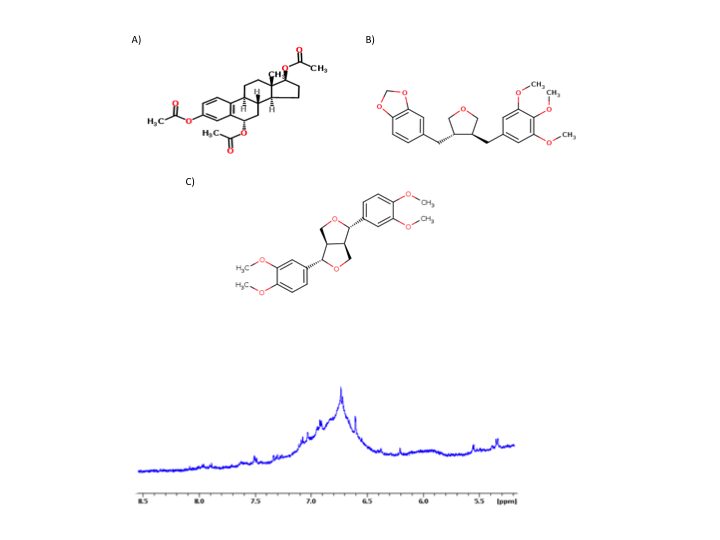

Supplement: S11 Fig — A) Estra-1,3,5(10)-triene-3,6beta,17beta-triol triacetate B) Burseran C) (+)Eudesmin D) Extended aromatic region of the NMR spectrum. (TIFF) [file pone.0177316.s017.tiff]

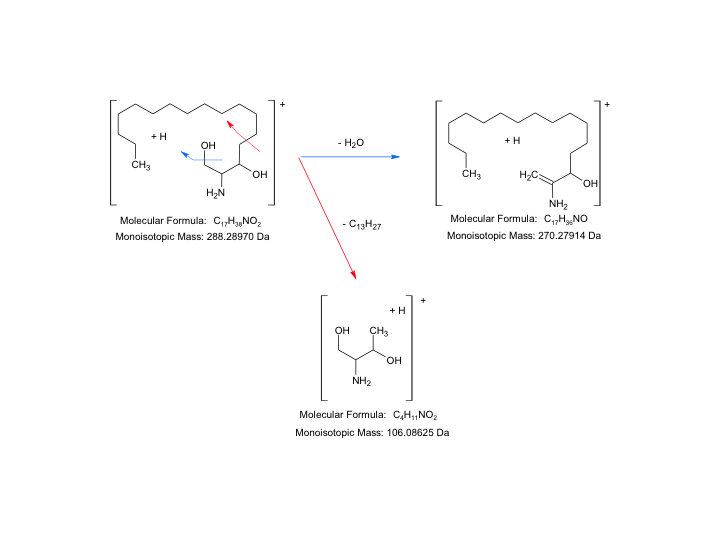

Supplement: S12 Fig — (TIFF) [file pone.0177316.s018.tiff]

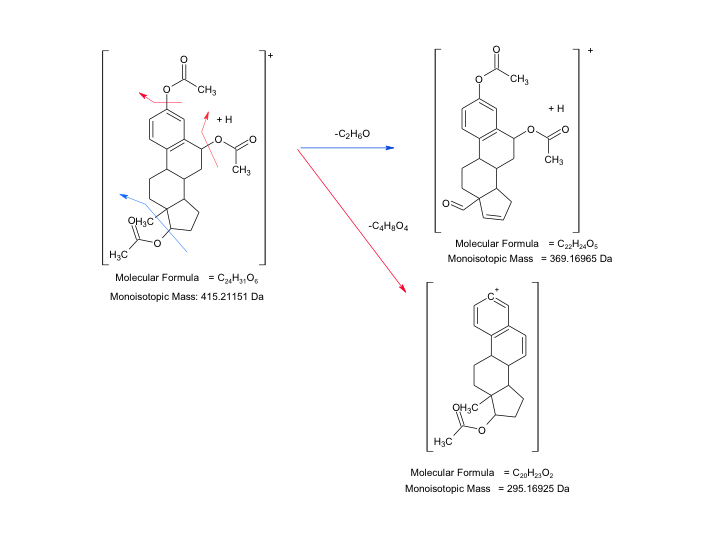

Supplement: S13 Fig — (TIFF) [file pone.0177316.s019.tiff]

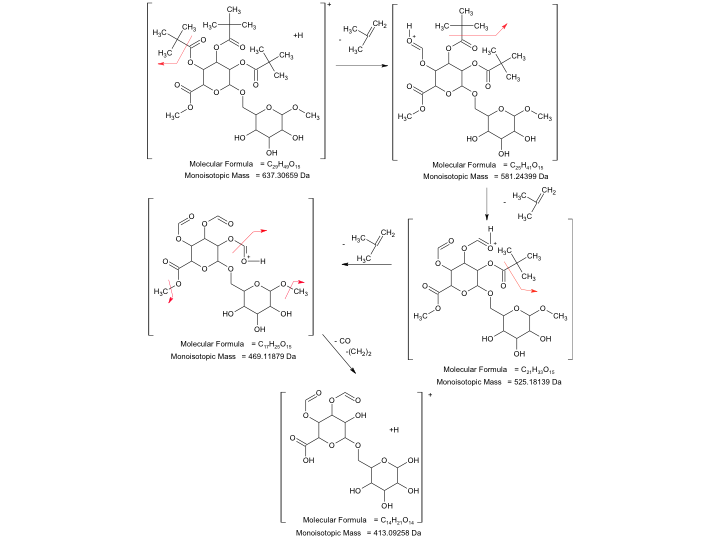

Supplement: S14 Fig — (TIFF) [file pone.0177316.s020.tiff]
